# Local environments, not invasive hybridization, influence cardiac performance of native trout under acute thermal stress

**DOI:** 10.1111/eva.13663

**Published:** 2024-02-22

**Authors:** Jeffrey T. Strait, Jared A. Grummer, Nicholas F. Hoffman, Clint C. Muhlfeld, Shawn R. Narum, Gordon Luikart

**Affiliations:** ^1^ Flathead Lake Biological Station, Wildlife Biology Program University of Montana Polson Montana USA; ^2^ Columbia River Inter‐Tribal Fish Commission Hagerman Idaho USA; ^3^ U.S. Geological Survey, Northern Rocky Mountain Science Center West Glacier Montana USA

**Keywords:** admixture, cardiac performance, climate change, heart rate, introgression, invasive hybridization, physiological stress, rainbow trout, thermal tolerance, Westslope cutthroat trout

## Abstract

Climate‐induced expansion of invasive hybridization (breeding between invasive and native species) poses a significant threat to the persistence of many native species worldwide. In the northern U.S. Rocky Mountains, hybridization between native cutthroat trout and non‐native rainbow trout has increased in recent decades due, in part, to climate‐driven increases in water temperature. It has been postulated that invasive hybridization may enhance physiological tolerance to climate‐induced thermal stress because laboratory studies indicate that rainbow trout have a higher thermal tolerance than cutthroat trout. Here, we assessed whether invasive hybridization improves cardiac performance response to acute water temperature stress of native wild trout populations. We collected trout from four streams with a wide range of non‐native admixture among individuals and with different temperature and streamflow regimes in the upper Flathead River drainage, USA. We measured individual cardiac performance (maximum heart rate, “MaxHR”, and temperature at arrhythmia, “ArrTemp”) during laboratory trials with increasing water temperatures (10–28°C). Across the study populations, we observed substantial variation in cardiac performance of individual trout when exposed to thermal stress. Notably, we found significant differences in the cardiac response to thermal regimes among native cutthroat trout populations, suggesting the importance of genotype‐by‐environment interactions in shaping the physiological performance of native cutthroat trout. However, rainbow trout admixture had no significant effect on cardiac performance (MaxHR and ArrTemp) within any of the three populations. Our results indicate that invasive hybridization with a warmer‐adapted species does not enhance the cardiac performance of native trout under warming conditions. Maintaining numerous populations across thermally and hydrologically diverse stream environments will be crucial for native trout to adapt and persist in a warming climate.

## 1 INTRODUCTION

Invasive species are among the greatest threats to native biodiversity worldwide (Allendorf et al., 2001; Grabenstein & Taylor, 2018; Rhymer & Simberloff, 1996). Human‐mediated species introductions can result in hybridization and genetic introgression with closely related native species, sometimes resulting in outbreeding depression (e.g., reduced fitness; Muhlfeld, Kalinowski, McMahon, et al., 2009; Muhlfeld, Mcmahon, Belcer, & Kershner, 2009; Muhlfeld, McMahon, Boyer, & Gresswell, 2009), ecological displacement of native species, and loss of native species diversity (Cucherousset & Olden, 2011; Gozlan et al., 2010). In extreme cases, hybridization can lead to a hybrid swarm and genomic extinction of native species (Allendorf et al., 2001; Cummins et al., 2023; Smith, 2023). Moreover, climate change can exacerbate the spread of invasive hybridization (Muhlfeld et al., 2014), so this problem will likely become more serious in the future (Arce‐Valdés & Sánchez‐Guillén, 2022; Kelly et al., 2010).

Despite the challenges posed to biodiversity conservation by invasive hybridization, it can lead to the creation of novel genotypes and allelic combinations that may confer selective advantages to admixed individuals in natural systems. Heterosis, or “hybrid vigor”, is one such advantage, where heterozygotes exhibit higher fitness than parental forms. Additionally, hybridization can generate novel phenotypes in terms of physiology, morphology, and behavior, promoting adaptation and potential range expansion in response to changing environmental conditions (Becker et al., 2013; Brauer et al., 2023; Kulmuni et al., 2023; Pfennig et al., 2016). However, some of these beneficial effects may diminish in advanced‐generation hybrids (Allendorf et al., 2022; Edmands, 1999; Muhlfeld, Kalinowski, McMahon, et al., 2009). Therefore, the outcomes of hybridization are multifaceted and often conflicting, necessitating further research to understand the evolutionary and ecological consequences of invasive hybridization in nature, especially considering the increasing threats of climate change and species introductions globally.

Climate‐induced range shifts can lead to increased sympatry between previously isolated species, potentially resulting in introgressive hybridization (Becker et al., 2013; Garroway et al., 2010; Kulmuni et al., 2023). This is particularly true for ectothermic species that are sensitive to temperature and streamflow conditions (Chunco, 2014; Hoffmann & Sgrò, 2011; Kelly et al., 2010). Hybridization and introgression are particularly common in salmonid fishes because there are limited pre‐ or post‐zygotic barriers to introgression among closely related taxa, and widespread stocking of non‐native species has created sympatry between previously allopatric species (Allendorf et al., 2001). Widespread introductions of non‐native rainbow trout (*Oncorhynchus mykiss*), the most widely introduced fish globally, have been particularly detrimental to all subspecies of inland cutthroat trout in western North America, resulting in widespread introgression that commonly leads to the formation of hybrid swarms and loss of native cutthroat genomes (Allendorf & Leary, 1988).

**FIGURE 1:**
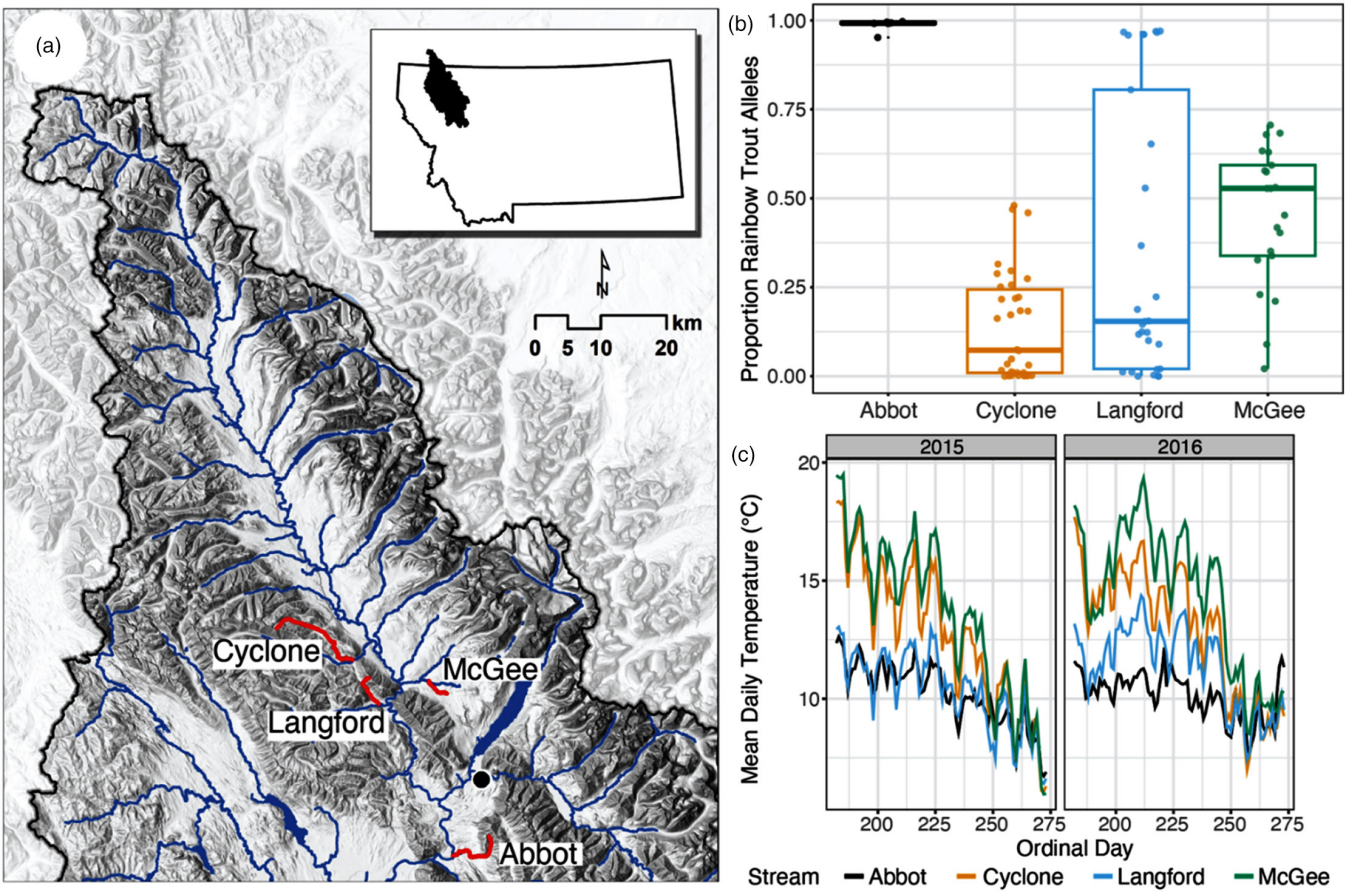
Map (a) showing streams sampled for this study in northwestern Montana, USA, along with the distribution of individual‐level rainbow trout admixture (b) by population (dots are individuals). Panel (c) shows recorded stream temperatures from ~late June until early October for 2015–2016. Boxplots in (b) depict median, first, and third quartiles (box bounds), and whiskers extend to 1.5× the interquartile range.

In the Flathead River system, a large and relatively pristine watershed in the Rocky Mountains of western North America, hybridization between native westslope cutthroat trout (*Oncorhynchus clarkii lewisi*) and introduced rainbow trout (with a divergence time of ~10 million years ago; Ma et al., 2016; Macqueen & Johnston, 2014) has been occurring over the past ~30+ years (Boyer et al., 2008; Hitt et al., 2003). Although approximately 20 million rainbow trout were stocked in this region beginning in the mid‐19th century (Muhlfeld et al., 2014), it was not until the late 1990s and early 2000s that rainbow trout alleles began to spread upstream from historical stocking locations (Boyer et al., 2008; Hitt et al., 2003; Muhlfeld et al., 2014). The primary contemporary source of rainbow trout alleles in this system is likely from a private hatchery located in the lower portion of the drainage (near Abbot Creek, Boyer et al., 2008; Muhlfeld, Mcmahon, Belcer, & Kershner, 2009; Figure 1). Previous research has shown that the percentage of westslope cutthroat–rainbow trout admixture decreases with distance from Abbot Creek (Hitt et al., 2003; Muhlfeld, McMahon, Boyer, & Gresswell, 2009), and that most rainbow trout alleles (85%) found in Abbot Creek are also found in hybridized populations throughout the watershed (Boyer et al., 2008). Together, these results indicate that North Fork Flathead River hybridized streams—including the streams studied here—share the same RBT source and therefore genotypes/alleles.

Rainbow trout hybridization in the Flathead system has spread rapidly over the past several decades due to interactions between changing climatic conditions (e.g., increased water temperatures and decreased spring streamflow) and historical stocking practices (Muhlfeld et al., 2014, 2017). Warming water temperatures, shifting streamflow regimes, and increasing frequency of extreme drought, flood, and wildfire events (Gervais et al., 2020; Isaak et al., 2012) are hypothesized to benefit rainbow trout, as rainbow trout tolerate warmer temperatures, lower spring flows, and greater environmental disturbance than cutthroat trout (Fausch et al., 2001; Muhlfeld, Kalinowski, McMahon, et al., 2009; but see Macnaughton et al., 2021).

Thermal adaptation, however, is complex in salmonid fishes with effects on many biological processes. Studies show evidence for shifts in physiological traits such as cardiac performance to deliver oxygen more efficiently to internal tissues as water temperature increases (e.g., Eliason et al., 2011). Furthermore, cardiac function has been observed to limit thermal tolerance of ectothermic organisms, including salmonid fishes (Christen et al., 2020; Ekström et al., 2019; Haverinen & Vornanen, 2020). In particular, salmonid populations that have evolved in warm climate regimes exhibit higher critical thermal maxima (CT_max_), maximum heart rate (*f*_h,max_ or “MaxHR”), and aerobic scope under stressful water temperatures than typical populations from cooler environments (e.g., Chen, Farrell, Matala, & Narum, 2018). While cardiac performance and thermal tolerance can be altered by acclimation temperatures in both rainbow trout (redband; *O. mykiss gairdneri*; Chen, Farrell, Matala, & Narum, 2018) and westslope cutthroat trout (Enders & Durhack, 2022)—which reflects plasticity within each species—evidence also exists for local adaptation in these traits among populations from distinct climates (e.g., Chen, Farrell, Matala, Hoffman, & Narum, 2018). A broader understanding of the physiological effects of hybridization and the potential role of local environmental conditions is needed to inform climate adaptation strategies and to protect native species threatened by invasive hybridization under future climate change.

Laboratory studies demonstrate that rainbow trout have higher survival, growth, and metabolic rates than westslope cutthroat trout at warmer temperatures (Bear et al., 2007; Rasmussen et al., 2012). Furthermore, candidate adaptive genes and genome regions for thermal tolerance have been reported in rainbow trout (Andrews et al., 2023; Chen & Narum, 2021). Therefore, hybridization between these species could result in the formation of novel genotypes that possess unique combinations of traits that improve thermal performance, such as heart function and cardiovascular performance (hereafter, “cardiac performance”). Additionally, populations within each of these salmonid species have become adapted to local environments with possible examples in both rainbow trout (e.g., Narum et al., 2013) and cutthroat trout (e.g., Amish et al., 2019). However, the physiological effects of hybridization on individual cardiac performance in response to warming temperatures remain untested.

Here, we investigated cardiac performance under acute thermal stress of native westslope cutthroat trout populations from streams with different temperature and streamflow regimes, which also had individuals of varying levels of invasive rainbow trout admixture. We hypothesized that local adaptation due to environmental conditions may be occurring in stream populations and that rainbow trout hybridization may improve thermal tolerance of hybrids compared to non‐hybridized cutthroat trout. Specifically, we quantified cardiac performance of westslope cutthroat trout and hybrids in response to increasing water temperatures using cardiac performance trials in a controlled laboratory environment. A collection of rainbow trout from a putative historical stocking location also provided a reference to compare differences in cardiac performance between the two parental species.

## 2 METHODS

### 2.1 Study area and field methods

We sampled *Oncorhynchus* spp. individuals during the summers of 2015 and 2016 in Cyclone, Langford, McGee, and Abbot (2015 only) Creeks in the North Fork Flathead River drainage in northwestern Montana, USA (Figure 1). Hourly water temperatures were recorded from May through September in each stream using Onset HOBO data loggers. These streams are primarily snowmelt driven, characterized by a peak discharge in the spring and warming temperatures throughout the summer months. Langford Creek is distinct from the other study streams because it has more groundwater and hyporheic flows and thus tends to be more thermally and hydrologically stable throughout the year (Figure 1c, Table S1). From the HOBO data, we calculated the maximum temperature and mean daily summer temperatures (from May to September) for each year and stream (Table S1).

Fish were sampled in June–July using backpack electrofishing by completing a single pass until 10–15 individuals were captured. For each individual, we measured total length (TL, mm) and mass (g). All individuals kept for cardiac testing weighed between 4 and 45 g (Figure S4) as a means to control for body size, which is known to influence heart rate (Altimiras & Larsen, 2000; Chen, Farrell, Matala, & Narum, 2018). Furthermore, mass was still corrected for when analyzing cardiac performance (see below).

Fish were transported to the U.S. Geological Survey (USGS) laboratory in West Glacier, Montana, and allowed to acclimate for approximately 12–24 h between 8 and 10°C before cardiac testing (as in Chen, Farrell, Matala, & Narum, 2018). After cardiac testing was concluded, fish were monitored for several hours and then released into the stream reach from which they were captured. From previous sampling efforts in these streams, we expected to encounter westslope cutthroat trout, rainbow trout, and hybrids, except in Abbot Creek, which contains rainbow trout and highly admixed individuals (Boyer et al., 2008).

### 2.2 Cardiac performance trials

We modified a method from previous studies (Casselman et al., 2012; Chen, Farrell, Matala, Hoffman, & Narum, 2018) to measure cardiac performance. Briefly, individual fish were anesthetized using 60–65 mg/L tricaine methanesulfonate (MS222) buffered with 160 mg/L NaHCO_3_ (Sigma‐Aldrich, St. Louis, MO, USA) after being held for 12–24 h at temperatures between 8 and 10°C. Prior to being placed in a custom‐built electrocardiogram (ECG) apparatus, a small tissue from the upper caudal fin was collected for genetic analysis. Due to logistical constraints of the sampling, we were unable to confirm the genetic ancestry of individuals prior to running cardiac performance trials. Each fish was acclimated for 30 min at 10°C while submerged in the ECG apparatus. After the 30‐min stabilization period, an intraperitoneal injection of 2.7 mg/kg atropine sulfate and 9 μg/kg isoproterenol was administered. This was followed by a 15‐min interval for the drugs to take effect and an elevated, stabilized heart rate (*f*_h,max_) was observed (Chen, Farrell, Matala, Hoffman, & Narum, 2018). After an elevated and stabilized *f*_h,max_ was observed, water temperature was increased 1°C every 6 min until peak *f*_h,max_ was reached. Determination of peak *f*_h,max_ (i.e., maximum heart rate) was identical to methods described by Chen, Farrell, Matala, Hoffman, and Narum (2018). Thus, we recorded the temperature at which each individual fish went arrhythmic, then calculated the proportion of fish from each stream that were arrhythmic at each temperature. Fish were recorded as such in both 2015 and 2016, and the data were combined across years within each stream for analyses (see Figures S1–S3 for measurements by year). Fish were then removed from the experiment and recovered before being released back into their respective stream of capture.

### 2.3 Genetic analyses

We genotyped individuals using the Rapture protocol (Ali et al., 2016) at 650 rainbow trout diagnostic loci that were distributed throughout the rainbow trout genome. The 95 samples used in this study were prepared in conjunction with those from Strait et al. (2021), following the same DNA extraction, Best‐Rad library preparation, RAD‐capture, and bioinformatic protocols to obtain each individual's genotypes as described below. We quantified the genome‐wide proportion of rainbow trout admixture (pRBT) for each individual using the rainbow trout diagnostic loci.

#### 2.3.1 Laboratory protocols

To calculate individual genome‐wide pRBT, we extracted genomic DNA using the SPRI bead extraction protocol described in Ali et al. (2016). The concentration of double‐stranded DNA was measured using QuantIt Picogreen assays (Thermo Fisher Scientific, Waltham, MA) after diluting samples to less than 20 ng/μL, and DNA quality (260/280 ratio) was measured using a Nanodrop 2000 spectrophotometer (Thermo Scientific).

Sequencing libraries were prepared using the bestRAD and Rapture (RAD capture) protocols (Ali et al., 2016) using 50 ng of input DNA for each sample. Libraries were sheared to an average fragment size of 350 base pairs using a Covaris E220 Ultrasonicator (Covaris Inc., Woburn, MA). Libraries were amplified for 12 cycles using a plate‐specific indexing primer, purified using Ampure XP beads, and quantified using Quantit Picogreen assays. Plates were pooled in groups of six (83 ng from each library) before enriching for 3015 RAD loci, which includes the ~650 RBT diagnostic loci that are informative for estimating admixture coefficients. Enrichment was performed using a custom Mybaits target enrichment kit (V3, Arbor Biosciences, Ann Arbor, MI). This panel included a combination of baits complementary to previously identified RAD loci containing westslope cutthroat trout polymorphic single‐nucleotide polymorphisms (SNPs) and rainbow, westslope cutthroat, and Yellowstone cutthroat trout species diagnostic SNPs (Amish et al., 2012; Hand et al., 2015; Hohenlohe et al., 2013; Kovach, Hand, et al., 2016). Loci were chosen for capture based on their genotyping quality, reliability for distinguishing each (sub)species, and even distribution across the assembled rainbow trout genome (Berthelot et al., 2014). Pooled libraries were amplified post‐capture for 10–12 cycles and quantified using Quantit Picogreen assays before being sequenced with 12 libraries per lane on an Illumina HiSeq X (Novogene Corporation, Sacramento, CA).

#### 2.3.2 Bioinformatics and genotype calling

Sequence read quality was evaluated using fastQC v0.11.5 (Andrews, 2010), and duplicate reads were removed using the *clone_filter* program from Stacks v1.44 (Catchen et al., 2011). Sequencing adapter contamination was removed from reads using *Trimmomatic* and reads were truncated whenever the mean Phred score across a window of four nucleotides dropped below q15. We required each read to be greater than 60 bp after applying the trimming steps above. Properly oriented fastq files were demultiplexed by individual barcodes using *process_radtags* from Stacks v1.44. Reads were mapped to the OmyK_1.0 rainbow trout reference genome (GCF_002163495.1; Berthelot et al., 2014) using bwa‐mem (Li, 2013) and resulting sam files were sorted, converted to bam format, and indexed using SAMtools v1.4 (Li et al., 2009). We then used HaplotypeCaller v3.7 (GATK) to generate gVCF files for each individual, combined gVCF files across individuals using CombineGVCFs v3.7, and called genotypes using GenotypeGVCFs v3.7 (McKenna et al., 2010). The resulting VCF file was filtered using VCFtools (LGPLv3; Danecek et al., 2011) as follows.

Genotypes were called missing if the genotype quality score was less than 30 and read depth was less than 7. In addition, an allele balance between 0.25 and 0.75 and a minimum read depth of 10 were required for all heterozygous genotypes. After filtering, loci were removed if they were missing genotypes in more than 10% of individuals, and individuals were removed prior to analysis if they did not have genotypes at more than 20% of all 3015 loci (i.e., not just RBT diagnostic loci). Proportion rainbow trout admixture (pRBT) was estimated for each individual as the number of rainbow trout alleles/(2*number of genotyped diagnostic loci). We genotyped 95 individuals (54 from 2015 and 41 from 2016) from all sites using 650 rainbow trout diagnostic loci (that remained after filtering). The median number of loci per individual was 536, and all individuals were genotyped at a minimum of 160 rainbow trout diagnostic loci.

### 2.4 Statistical analyses

We tested for the effects of pRBT and environmental conditions on mass‐corrected peak heart rate *f*_h,max_ (maximum heart rate, MaxHR) and temperature at arrhythmia (ArrTemp). We performed a mass correction for heart rate following previous studies with fish of similar age and size as we used here (i.e., Chen, Farrell, Matala, & Narum, 2018) because heart rate is affected by the mass of the individual (Chen, 2017). The mass‐corrected heart rate was obtained with the following equation: (measured heart rate)*((weight (g)/11.8)^0.1^). For statistical analyses, we only included populations that exhibited a wide range of individual pRBT to allow testing for individual effects of admixture (pRBT) within streams (environmental or population level) and also for differences in cardiac performance among streams. Thus, Abbot Creek was not used in model testing since it lacks sufficient variation in individual‐level pRBT (Figure 1b). Nonetheless, individuals from Abbot Creek serve as a reference in the study region for nearly non‐admixed rainbow trout, and the inclusion of Abbot Creek did not change our model testing results (see below).

The goal of this analysis was to understand if cardiac performance was best explained by individual proportion rainbow trout admixture (pRBT), differences in local environmental conditions (stream or year), or both. To achieve this goal, we conducted linear mixed‐effects modeling using the package *lme4* in R (Bates et al., 2015). Due to fairly small sample sizes, we did not test for measurable environmental conditions (e.g., temperature) as explanatory variables on cardiac performance. Instead, we allowed for spatial (|Stream) or temporal (|Year) variation to be absorbed into random effects. However, due to issues of overfitting the models, we could not test for both |Stream and |Year simultaneously, and thus tested for these effects separately. Prior to linear modeling, pRBT was logit transformed (lpRBT) as it is a proportion and not normally distributed. We included the following models in our model selection process:

Cardiac Performance~∣Stream;

Cardiac Performance~lpRBT+∣Stream;

Cardiac Performance~∣Year;

Cardiac Performance~lpRBT+∣Year;

where the response variable for cardiac performance was MaxHR or ArrTemp. We used the Akaike information criterion corrected for small sample sizes (AICc) to rank the relative support for each model, with a ΔAICc of >2 indicating significant difference between models. Finally, we used two‐way analysis of variance (ANOVA) to discern between competing, highly ranked models.

## 3 RESULTS

During the summer, all study streams were relatively cool and characterized by maximum temperatures between ~10 and 17°C (Figure 1c, Table S1). McGee was the warmest stream overall (~22°C), whereas Langford was the coolest (~16.5°C). Abbot had the highest variation in interannual maximum summer temperatures (14.1–20.9°C), whereas Cyclone (19.9–21.2°C) and Langford (15.7–17.0°C) experienced more stable maximum summer temperatures (Table S1).

### 3.1 Hybridization characterization

We genotyped 95 individuals for 650 rainbow trout diagnostic loci collected from wild populations (see Table 1 for sample sizes). Abbot Creek contained nearly all non‐admixed rainbow trout individuals (median pRBT = 0.99), whereas Cyclone Creek had the lowest proportion of rainbow trout admixture (mean pRBT = 0.14; Figure 1b). Abbot Creek lacked sufficient variation in proportion rainbow trout admixture (Figure 1b) to be able to separate admixture effects from local stream effects (i.e., environment effects or local adaptation) and thus was not included in statistical analyses. Nonetheless, the non‐hybridized rainbow trout collected from Abbot Creek provided a useful reference for comparison to non‐hybridized westslope cutthroat trout. We present Abbot Creek results below for comparisons with Cyclone, Langford, and McGee Creeks, which had a wide range of admixed individuals (e.g., Langford Creek individuals ranged in pRBT from 0.00 to 0.97; Figure 1b). See Figure S5 for pRBT distributions of individuals from this study as compared to a larger number of samples from the same study populations.

**TABLE 1:** Summary of admixture and cardiac performance metrics for each stream and year sampled (along with the non‐hybridized rainbow trout reference stream, Abbot) in Montana, USA.

Stream	Year	Sample size	Mean pRBT	Minimum pRBT	Maximum pRBT	Mean weight (g)	Mean ArrTemp (°C)	Mean MaxHR (bpm)	Mean mass‐Corrected MaxHR
Abbott	2015	6	0.987	0.952	0.997	7.17 (1.19)	25.52 (0.68)	186.4 (6.49)	225.15 (5.86)
Cyclone	2015	21	0.1	0.001	0.469	8.95 (0.51)	17.7 (0.76)	118.93 (5.55)	147.29 (6.63)
Cyclone	2016	13	0.206	0	0.479	12.85 (11.07)	18.18 (0.49)	128.99 (3.54)	166.22 (5.74)
Langford	2015	12	0.056	0	0.146	12.00 (2.21)	21.03 (0.61)	143.87 (4.63)	181.45 (6.09)
Langford	2016	16	0.62	0.1	0.97	12.88 (0.78)	18.86 (0.53)	126.96 (4.69)	163.64 (6.32)
McGee	2015	9	0.38	0.021	0.633	9.89 (0.77)	19.57 (0.96)	114.88 (7.22)	144.18 (9.1)
McGee	2016	12	0.507	0.089	0.706	13.25 (0.95)	16.75 (0.78)	110.38 (4.91)	142.85 (7.01)

*Note*: Admixture statistics include the sample mean, minimum, and maximum proportion rainbow trout admixture (pRBT) in sample collections (based on approximately 650 rainbow trout diagnostic single‐nucleotide polymorphism (SNP) loci). Relevant to cardiac performance, we included mean weight (grams), mean arrhythmia temperature (ArrTemp, °C), mean maximum heart rate (MaxHR, beats per minute), and the mass‐corrected peak maximum heart rate (mass‐corrected MaxHR~[MaxHR*[weight/11.8]]^0.1^). The standard error of each metric is included in parentheses. See Figures 1, 2, 3 and Figure S4 for the full distributions of these statistics.

### 3.2 Cardiac performance

**FIGURE 2:**
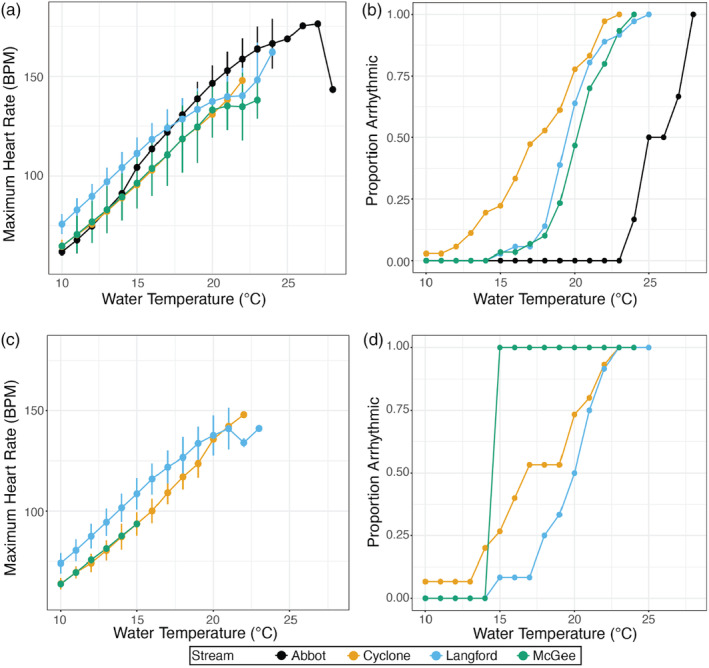
Mass‐corrected maximum heart rate (mean and standard error; see main text for conversion equation) as a function of water temperature (a, c), and the proportion of individual trout experiencing arrhythmia at each recorded temperature (b, d) for four streams in Montana, USA. Panels (a and b) show data for all individuals, whereas (c and d) show results only for individuals with less than 10% genome‐wide rainbow trout alleles (pRBT < 0.1).

We found significant differences in cardiac performance among individuals from different streams. When considering all individuals (e.g., pRBT 0.0–1.0), individuals from Langford Creek had the highest resting heart rate at lower temperatures, whereas Abbot Creek individuals had the lowest (Figure 2a). However, at the warmer temperatures (18+°C), Abbot Creek individuals showed the highest MaxHR (peak *f*_h,max_) and were able to tolerate warmer temperatures beyond the other populations. All Cyclone Creek individuals experienced arrhythmia by 23°C, whereas Abbot Creek individuals did not begin to experience arrhythmia until 23°C, and all Abbot Creek individuals did not become arrhythmic until 28°C (Figure 2b). Furthermore, individuals from Cyclone Creek showed the highest variation in MaxHR, where individuals experienced arrhythmia between 10 and 23°C. All individuals from the three focal streams (Cyclone, Langford, and McGee Creeks) experienced arrhythmia between 23 and 25°C (Figure 2b).

**FIGURE 3:**
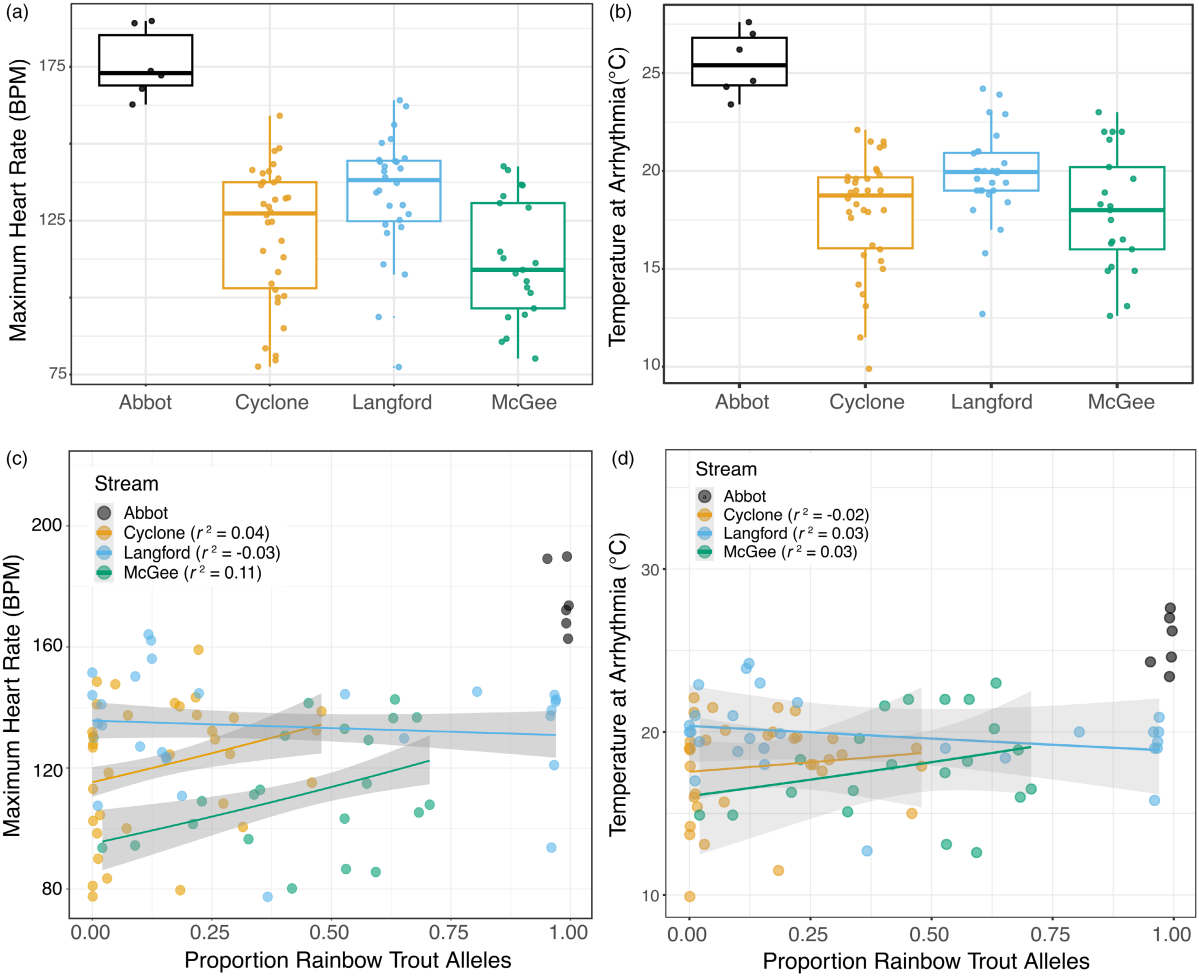
Distributions of mass‐corrected maximum heart rate (peak; a) and temperature at arrhythmia (ArrTemp, b) by populations in four streams in Montana, USA. Both linear mixed‐effects models and ANOVA results support the variation in MaxHR and ArrTemp are due to stream effects (*p* < 0.03; Tables S2 and S3). Panels (c and d) show maximum heart rate and maximum temperature achieved at arrhythmia as a function of proportion of rainbow trout admixture (proportion of alleles from rainbow trout ancestry, “pRBT”), respectively. The best‐supported linear mixed‐effects models did not include an effect of pRBT on cardiac performance. To illustrate the weak and variable effect of pRBT on cardiac performance, we plotted lines fit with generalized linear models (shading represents model standard error) and *r*‐squared correlations indicated in legends; none of the relationships were statistically significant (*p* > 0.08). Abbot Creek had low interindividual pRBT variation and too few individuals (*n* < 7) to conduct statistical analyses, so they were removed from statistical analyses, and shown here only for reference.

When comparing individuals with low versus high rainbow trout admixture within a population, relationships between heart rate (*f*_h,max_) and water temperature were largely similar (e.g., Figure 2a vs. c). McGee Creek showed the largest differences between low‐ and high‐admixed fish; low‐ and non‐admixed westslope cutthroat trout were all arrhythmic by 15°C (Figure 2d), whereas more highly admixed fish did not become arrhythmic until 23°C (Figure 2b). In spite of the overall similarities, higher proportions of low‐admixed individuals (pRBT < 0.1) were arrhythmic at lower temperatures as compared to all individuals (Figure 2b vs. d). Furthermore, even among individuals with little or no rainbow trout admixture (pRBT < 0.1), we found a large variation in cardiac performance (e.g., arrhythmia temperature ranged from 9.9 to 23.9°C across streams; Figure 3b). Overall, we documented large variation in cardiac performance both within and among the three focal populations (e.g., excluding Abbot Creek reference population), and this high variation was found across individuals of all admixture proportions (Figure 3).

We found stronger differences in cardiac performance among streams than among admixture proportions. Large differences existed in both the mean and variance of cardiac performance among streams (Figure 3a,b). Despite the apparent (weak) effects of individual admixture, streams exhibited variable, distinct, and significant effects on cardiac responses (*p* > 0.08; Figure 3c,d). Because the top‐ranked model only included a stream effect, we visualized the relationship between pRBT and cardiac performance in Figure 3 with a generalized linear model for each stream. Individuals from within Cyclone and McGee Creeks showed weakly positive relationships between pRBT and cardiac performance, but Langford Creek individuals exhibited weakly negative relationships between admixture and both maximum heart rate and temperature at arrhythmia (Figure 3c,d); these trends still hold if the Langford Creek individuals with pRBT > 0.75 are removed from the analysis to make this population's pRBT distribution (among individuals) more similar to that of Cyclone and McGee Creeks (Figure S6).

### 3.3 Linear modeling

Linear model results showed no significant evidence that rainbow trout admixture influenced cardiac performance. The inclusion of pRBT as a covariate in models did not result in a higher AICc rank than models containing only a random effect for stream for either MaxHR or ArrTemp (Table 2). For MaxHR, the model with pRBT and |Stream had the lowest AICc (AICc = 742.43; Table 2a). However, this model was less than two AICc points lower than the model that only contained a random effect for stream (∆AIC = 1.11), suggesting pRBT was adding little explanatory value to the model. A two‐way analysis of variance (ANOVA) supported these results, as a stream effect was statistically significant (*p* < 0.002) and pRBT was not supported (*p* > 0.35; Table S2).

**TABLE 2:** Candidate models of cardiac performance of fish sampled from Cyclone, Langford, and McGee Creeks in Montana, USA, in 2015 and 2016.

Covariate structure	Number of parameters	AICc	Δ AICc	Model likelihood	AICc weight
(a) Maximum heart rate					
pRBT + |Stream	4	742.43	0	1	0.62
|Stream	3	743.54	1.11	0.58	0.36
pRBT + |Year (*)	4	749.87	7.44	0.02	0.02
|Year (*)	3	750.76	8.33	0.02	0.01
(b) Temperature at arrhythmia					
|Stream	3	413.95	0	1	0.65
|Year	3	416.66	2.71	0.26	0.17
pRBT + |Stream	4	417.67	3.72	0.16	0.1
pRBT + |Year	4	417.99	4.04	0.13	0.09

*Note*: Models are ranked using Akaike information criterion corrected for small sample sizes (AICc). For each model, the covariate structure, number of parameters, AICc, ΔAICc, model likelihood, and AICc weight are reported. All values were computed using the *AICcmodavg* package in R. Panel (a) shows those results for models of mass‐correct maximum heart rate and panel (b) for those of temperature at arrhythmia. (*) Model overfit—should not be considered during selection.

We found similar results when evaluating cardiac performance with temperature at arrhythmia (ArrTemp) as the response variable. Specifically, the model containing only a random effect for stream was the top model supported via AICc (weight = 0.65; Table 2b). The highest‐ranking model containing pRBT as a covariate was >3.5 AICc points higher (AIC weight = 0.1) than the model with only stream effects (Table 2b). This result was further supported by a two‐way ANOVA, where pRBT was not significant (*p* > 0.48; Table S3). Full summaries of the top supported mixed‐effects linear models are included in the Supplemental Materials (Tables S4 and S5).

## 4 DISCUSSION

Invasive hybridization between invasive and native species is predicted to increase due to climate‐induced expansion of introduced species, with irreversible evolutionary consequences for native species and biodiversity. Across the northern Rocky Mountains of the United States and Canada, hybridization between native cutthroat trout and introduced rainbow trout has spread rapidly during a 30‐year period of accelerated warming (Bell et al., 2021; Muhlfeld et al., 2014, 2017), suggesting that invasive hybridization may enhance physiological tolerance of hybrid trout to climate‐induced thermal stress. Here, we tested whether invasive hybridization with non‐native rainbow trout enhances cardiac performance of native cutthroat trout under acute temperature stress. Our results revealed that, despite significant variation in cardiac performance among populations and experimental temperatures, rainbow trout admixture had no significant effect on cardiac performance (MaxHR and ArrTemp). Rather, we observed differences among streams suggesting possible genotype‐by‐environment effects where stream‐specific local environmental conditions played an important role in influencing cardiac performance during acute thermal stress (increasing temperature).

We did not detect a significant effect of hybridization on the cardiac performance in our three study streams, which is contrary to our hypothesis that invasive hybridization may enhance physiological tolerance of hybrid trout to thermal stress. Moreover, individuals from a stream with highly admixed individuals (Abbot Creek) had higher cardiac performance than all other fish in our study. Importantly, it is believed that all rainbow trout ancestry in this system derives from a single source ~30 years ago (Boyer et al., 2008; Hitt et al., 2003). Rainbow trout have one of the highest upper‐temperature tolerances among salmonid species (Pandey et al., 2021; but see Macnaughton et al., 2021). In our data (Figure 2) and those of (Bear et al., 2007), rainbow trout relative to westslope cutthroat trout had a nearly 5°C higher upper lethal temperature and greater resistance to prolonged exposure to temperatures above 20°C.

The effects of hybridization on cardiac performance, however, are poorly understood and limited in nature. Cooke and Philipp (2005) studied the cardiac response of largemouth bass (*Micropterus salmoides*) first‐generation hybrids and found that interstock hybrids (i.e., from intraspecific hybridization) had reduced cardiovascular recovery after exercise and higher resting levels of cardiac output and heart rate as compared to locally adapted native stocks. Beyond cardiac thermal performance in hybrids, a few studies have investigated the relationship between hybridization and thermal tolerance. Christen et al. (2020) studied Arctic and brook char (*Salvelinus alpinus* and *S. fontinalis*) and found that hybrids were not different or were intermediate in temperature tolerance (CT_max_) to parental types. Similarly, Wells et al. (2016) found no heterosis or difference in thermal tolerance between non‐admixed versus hybrid brook trout (*S. fontinalis*). Outside of Salmonidae, hybrids between redbelly dace (*Chrosomus erythrogaster*) and redside dace (*Clinostomus elongatus*) were generally intermediate‐to‐parental types (Turko et al., 2020); the median thermal tolerance (CT_max_) among redbelly dace, redside dace, and hybrids were 33.5, 34.9, and 34.7, respectively (see fig. 2 in Turko et al., 2020). Clearly, additional work would improve our understanding of the physiological effects of hybridization on native species, particularly in light of increasing human‐mediated species introductions and global climate change.

Several potential reasons could explain the lack of a hybridization effect on cardiac performance observed in this study. First, it is possible that genetic combinations resulting from hybridization did not confer significant advantages in cardiac function. While rainbow trout are known for their relatively high thermal tolerance (e.g., Chen et al., 2015), the genetic traits responsible for this characteristic might not have been effectively transferred or expressed in hybrid individuals of our study populations. Hybrids can suffer reduced fitness from intrinsic outbreeding depression or non‐native alleles, and multiple lines of evidence exist for outbreeding depression in hybrid cutthroat x rainbow trout, including reduced reproductive success (Muhlfeld, Kalinowski, McMahon, et al., 2009; Muhlfeld, Mcmahon, Belcer, & Kershner, 2009; Muhlfeld, McMahon, Boyer, & Gresswell, 2009), selection against juveniles (*s* = 0.6; Kovach, Hand, et al., 2016; Kovach, Luikart, et al., 2016), and genome‐wide selection against non‐native rainbow trout alleles (Kovach, Hand, et al., 2016; Kovach, Luikart, et al., 2016). Furthermore, mitochondrial genomes have evolved in their specific nuclear background, and disruption of mitonuclear coadaptation could contribute to oxidative stress in hybrids and ultimately hybrid breakdown in this study (e.g., Pichaud et al., 2019).

We found significant variation in cardiac response to temperature stress in native cutthroat trout among diverse stream environments. This finding is consistent with other salmonid species that exhibit genotype‐by‐environment interactions and adaptation to local thermal regimes including improved cardiac performance in populations that have experienced exposure to high water temperatures (Anttila et al., 2014; Chen, Farrell, Matala, Hoffman, & Narum, 2018; Chen, Farrell, Matala, & Narum, 2018; Eliason et al., 2011). However, populations of westslope cutthroat trout studied here displayed patterns of cardiac performance distinct from previous salmonid studies, where other salmonids exhibited improved performance through adaptation to extremely warm climates (e.g., Chen, Farrell, Matala, Hoffman, & Narum, 2018; Chen, Farrell, Matala, & Narum, 2018; Eliason et al., 2011).

Cutthroat trout and hybrids in Langford Creek displayed the widest range and highest cardiac performance relative to the other populations from warmer and more variable environments. Importantly, Langford individuals with high pRBT did not exhibit similar cardiac performance to those from Abbot Creek, nor did they perform significantly better than other Langford individuals with low pRBT. Groundwater and hyporheic flows in Langford Creek buffer stream temperatures from other climatic variables that typically drive local water temperatures (e.g., elevation, air temperature, and flow; Narum et al., 2013), leading to a more stable environment compared to the other study streams. Thermal stability, which can occur at multiple temporal scales (such as diel, seasonal, or annual; Kefford et al., 2022), is predicted to lead to a narrower thermal breadth (the difference between an organism's critical thermal minimum and maximum; e.g., Janzen, 1967). However, where the thermal variation is experienced in an organism's thermal performance curve is a key factor in determining the effect of thermal variation on physiological performance (Morash et al., 2018). Given that the temperature preference of westslope cutthroat trout is near 20°C (Macnaughton et al., 2021), the higher temperatures experienced in Langford Creek (or any of our study streams) are not likely to put fish into physiological stress (see Table S1). Because maximum summer temperatures in the three focal streams did not exceed the thermal preference range of cutthroat trout (Macnaughton et al., 2021), it is possible that the physiological benefits (e.g., adaptation) of experiencing stressful temperatures and/or wide ranges in seasonal (or daily) temperatures as described by Chen, Farrell, Matala, and Narum (2018) and Chen, Farrell, Matala, Hoffman, and Narum (2018) are not realized in these populations.

The individuals from Abbot Creek with very high pRBT had better cardiac performance than all admixed individuals that we tested, including individuals from Langford Creek with similar admixture levels. Abbot Creek also had high variation in maximum summer temperatures, which could contribute to its individuals' higher cardiac performance. This suggests Abbot Creek rainbow trout can serve as a reference point for the higher cardiac capacity of rainbow trout in this system compared to native westslope cutthroat trout and hybrids between these species. This result was not unexpected, as rainbow trout are known to have a higher CT_max_ and wider thermal tolerance than cutthroat trout (Bear et al., 2007). However, the cardiac performance of the Abbot Creek fish was greater than even that of desert‐adapted wild rainbow trout in Idaho (Chen, Farrell, Matala, Hoffman, & Narum, 2018; Chen, Farrell, Matala, & Narum, 2018), but this is consistent with the high thermal tolerance of coastal lineage rainbow trout (Chen et al., 2015). With the very low interindividual variation in pRBT within the Abbot Creek population, we can only speculate if these differences in cardiac performance were due to genetic differences between species or local adaptation and stream conditions. Where we did find westslope cutthroat trout sympatric with admixed rainbow trout (up to 0.97 pRBT), there was no difference in cardiac performance. In fact, fish of high admixture (>0.9 pRBT) from Langford Creek did not perform near the level of those from Abbot Creek, and fish of relatively low admixture were the closest in cardiac performance to Abbot Creek (Figure 3c,d).

While we found no evidence of differences in cardiac performance as a function of admixture, the interpretation of our results is fairly limited in scope. For example, sample sizes were relatively small, and disentangling the effects of non‐native admixture from differences among streams (local effects) is difficult. Common garden approaches and more extensive sampling are needed to understand the relative role of each effect. Additionally, we only considered the effects of proportion admixture (genome‐wide); however, genomic approaches (such as genome‐wide association tests) that use more genomic information (candidate genes and higher‐density markers) may reveal single‐ or multi‐locus effects of rainbow trout alleles on cardiac performance that were not detected in this study. Future research could include larger sample sizes, wider ranges of admixture (pRBT) within streams, sites with wider temperature ranges, and genetic data with higher genomic resolution—including mitochondrial DNA markers to test for mitonuclear disruptions, and also test for gene expression variation associated with admixture, as heritable expression variation (and plasticity) has been associated with thermal tolerance in fish (e.g., Li et al., 2017).

The results of our study revealed significant variation in cardiac performance among individual trout when exposed to thermal stress, particularly for non‐hybridized native westslope cutthroat trout from different environments. This finding suggests that individual‐level factors, such as genetic variability and phenotypic traits, likely play a key role in shaping the physiological response of native trout to temperature changes within populations. Additionally, the observed differences in cardiac response among populations occupying different stream environments highlight the likely influence of local adaptation to environmental conditions. This suggests that cutthroat trout populations in distinct stream habitats may possess specific physiological adaptations that allow them to cope differently with temperature stress. In large interconnected systems (e.g., metapopulations), this variation in physiological adaptations can help to ensure that populations can continually adapt to changing local environments via gene flow (Schindler et al., 2010; Thorson et al., 2018). More work would help us better understand the mechanisms influencing cardiac performance at both the individual and population levels and across diverse environmental conditions (Christen et al., 2020).

Overall, our results suggest that hybridization and introgression from a warmer‐adapted species did not improve the cardiac performance of native trout under acute thermal stress. Although natural hybridization may produce novel genotypes, invasive hybridization may be generally deleterious (genome wide) and may not promote adaptation to climate warming in cutthroat trout even if certain rainbow trout‐introgressed alleles are thermally adaptive within rainbow trout (Hamilton & Miller, 2016; Kovach, Luikart, et al., 2016; Perry et al., 2005 – see also Kardos & Shafer, 2018). Indeed, hybrid trout in this stream system have reduced fitness relative to non‐hybridized cutthroat trout (Allendorf et al., 2004; Muhlfeld, Kalinowski, McMahon, et al., 2009; Muhlfeld, Mcmahon, Belcer, & Kershner, 2009; Muhlfeld, McMahon, Boyer, & Gresswell, 2009). Recent authors have suggested using hybridization as a tool to improve fitness and population persistence (Chan et al., 2019; Hirashiki et al., 2021). However, many other authors caution against hybridization as a management tool when parental populations are highly divergent—for example, isolated hundreds of generations ago (Chan et al., 2019; Frankham et al., 2011; Kovach, Luikart, et al., 2016).

Our study provides evidence that invasive hybridization does not improve cardiac performance of native trout in the wild. Protecting genetic integrity and population diversity—which are critical for long‐term adaptation and resilience—will be important for native species threatened with climate‐induced invasive hybridization. Furthermore, eradicating sources of introgression and protecting non‐hybridized populations may be effective conservation strategies for adaptation and persistence of native species threatened with climate‐induced invasive hybridization. Maintaining multiple populations in diverse environments, connected via interstream migration, as here, can facilitate species' persistence.

## CONFLICT OF INTEREST STATEMENT

We declare we have no competing interests.

## Supporting information


Data S1:

## Data Availability

The datasets used in these analyses can be found at Dryad: http://doi.org/10.5061/dryad.crjdfn3c4.
