# Supplementary material for: Local environments, not invasive hybridization, influence cardiac performance of native trout under acute thermal stress
Source: Evol Appl. 2024 Feb 22;17(2):e13663. doi: 10.1111/eva.13663 (PMC10883762; doi:10.1111/eva.13663)
Supplement: Supplementary file 1 — Data S1: [file EVA-17-e13663-s001.docx]

Supplemental materials for “Local environments, not invasive hybridization, influence cardiac performance of native trout under acute thermal stress”, by Strait and Grummer *et al.*

Any use of trade, firm, or product names is for descriptive purposes only and does not imply endorsement by the U.S. government.


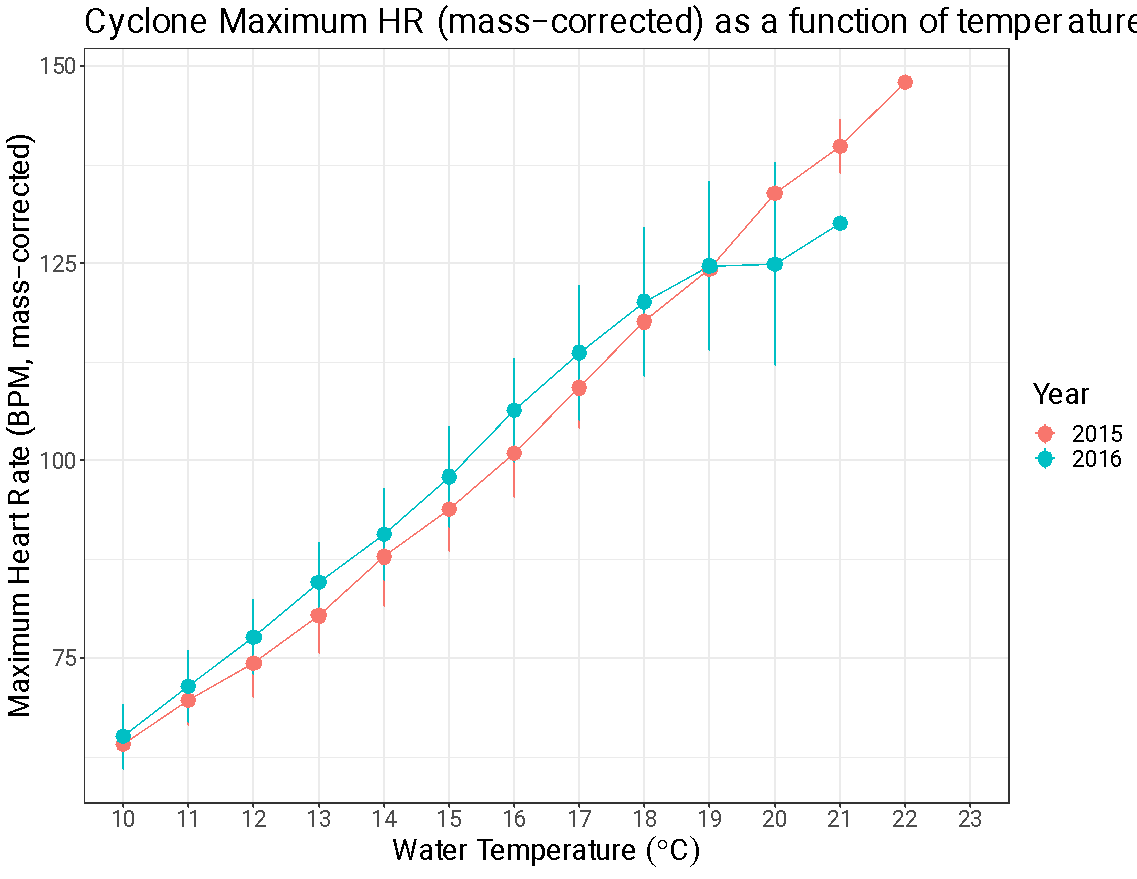


Figure S1. Maximum heart rate of individuals from Cyclone Creek as a function of temperature, with measurements colored by study year.


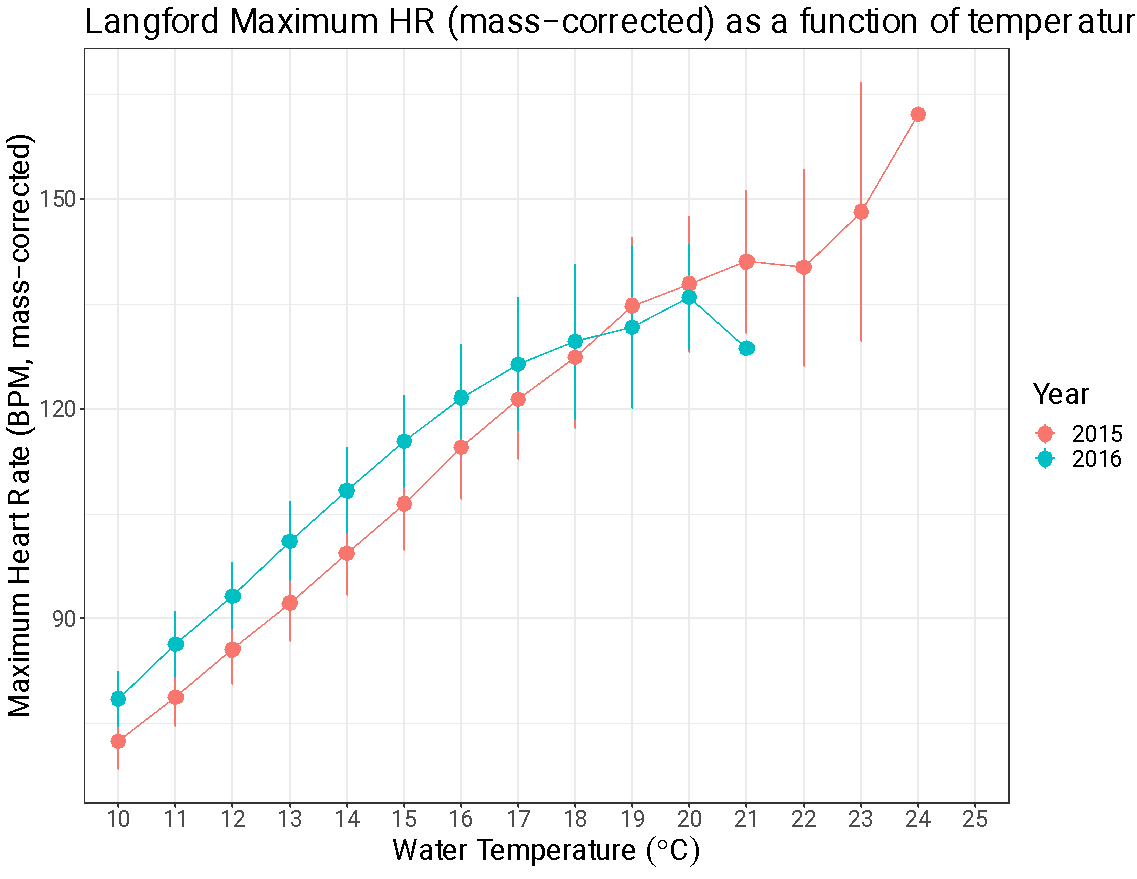


Figure S2. Maximum heart rate of individuals from Langford Creek as a function of temperature, with measurements colored by study year.


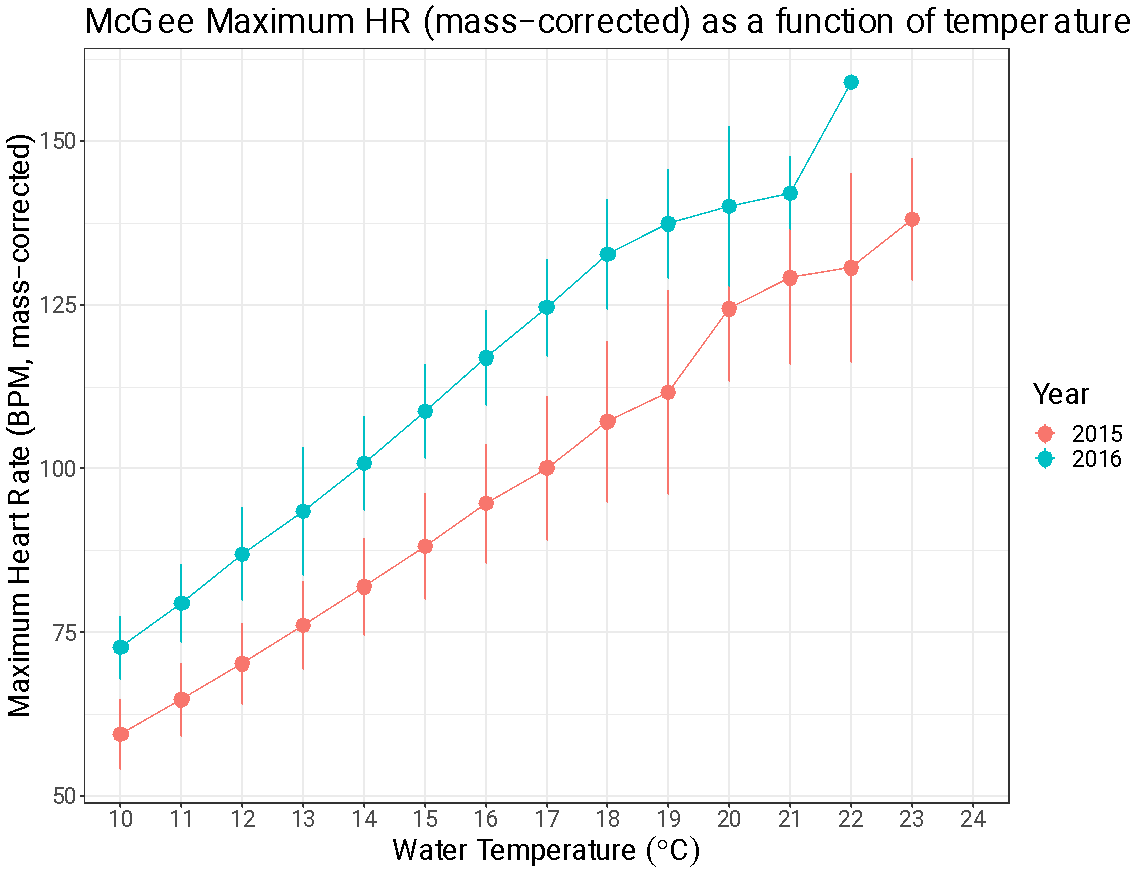


Figure S3. Maximum heart rate of individuals from McGee Creek as a function of temperature, with measurements colored by study year.


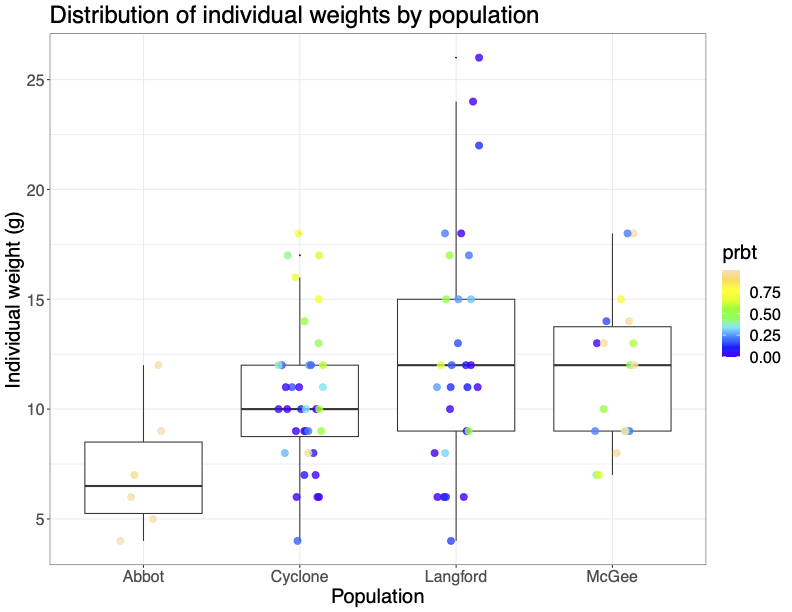


Figure S4. Distribution of weights of individuals in this study per population, with each individual colored by its proportion of rainbow trout alleles (pRBT).


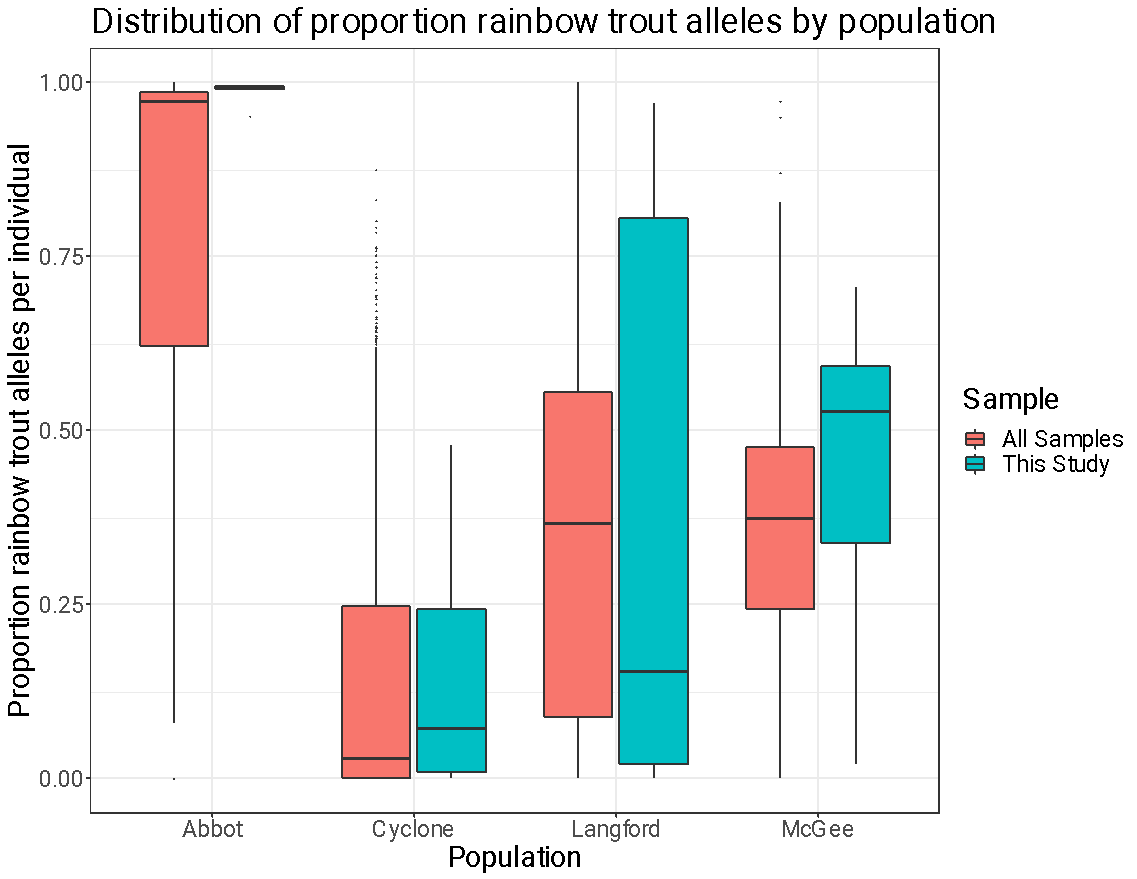


Supplemental Figure S5. Distributions of the proportion of rainbow trout alleles per individual for individuals measured in this study (teal) vs. a broader sampling of the populations (red).


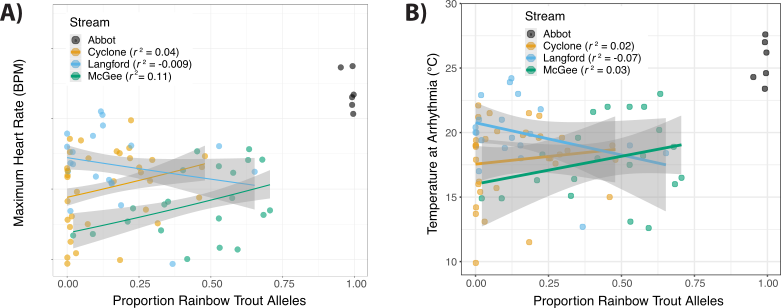


Figure S6. Maximum heart rate and temperature at arrhythmia as a function of admixture (proportion rainbow trout alleles; pRBT), when Langford individuals with pRBT > 0.75 are removed. Lines represent linear model fit and shading represents model standard error.


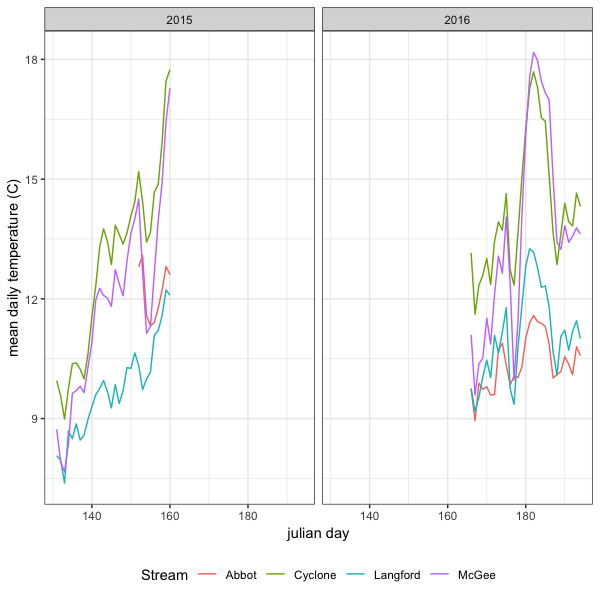


Figure S7. The recorded stream temperatures from each stream during the ~ one month prior to sampling (from mid-May to mid-June) from 2015-’16.

Table S1. Stream temperature (°C) data from HOBO dataloggers from 2013-2017. Maximum and mean summer temperatures (℃) were calculated from the hourly-recorded data between May 1 to September 30.

| **Stream** | **Year** | **Maximum summer temperature (℃)** | **Mean summer temperature (℃)** |
| --- | --- | --- | --- |
| Abbot | 2013 | 17.6 | 11.6 |
|  | 2014 | 20.9 | 12.7 |
|  | 2015 | 15.5 | 9.9 |
|  | 2016 | 14.1 | 9.7 |
|  | 2017 | 17.1 | 11.6 |
| Cyclone | 2013 | 21.2 | 12.3 |
|  | 2014 | 21.0 | 11.5 |
|  | 2015 | 19.9 | 11.3 |
|  | 2016 | 20.0 | 11.1 |
|  | 2017 | 19.9 | 10.8 |
| Langford | 2013 | 15.7 | 9.9 |
|  | 2014 | 15.9 | 9.9 |
|  | 2015 | 16.9 | 10.5 |
|  | 2016 | 17.0 | 11.2 |
|  | 2017 | 17.0 | 11.0 |
| McGee | 2013 | 21.9 | 13.2 |
|  | 2014 | 21.6 | 12.6 |
|  | 2015 | 22.0 | 13.0 |
|  | 2016 | 21.7 | 13.2 |
|  | 2017 | 20.5 | 11.4 |

Table S2. Summary of two-way Analysis of Variance (ANOVA) of maximum heart rate (MaxHR) by proportion rainbow trout admixture (pRBT) and stream.

| **Term** | **df** | **Sumsq** | **Meansq** | **Statistic** | ***p*-value** |
| --- | --- | --- | --- | --- | --- |
| lpRBT | 1 | 344.0751 | 344.0751 | 0.8108288 | 0.370612146 |
| Stream | 2 | 6,015.3584 | 3,007.6792 | 7.0877343 | 0.001475222 |
| Residuals | 79 | 33,523.6406 | 424.3499 |  |  |

Table S3. Summary of two-way Analysis of Variance (ANOVA) of arrhythmia temperature (ArrTemp) by proportion rainbow trout admixture (pRBT) and stream.

| **Term** | **df** | **Sumsq** | **Meansq** | **Statistic** | ***p*-value** |
| --- | --- | --- | --- | --- | --- |
| lpRBT | 1 | 3.83568 | 3.83568 | 0.4900456 | 0.48596337 |
| Stream | 2 | 61.56307 | 30.78153 | 3.9326417 | 0.02354201 |
| Residuals | 79 | 618.34800 | 7.82719 |  |  |

Table S4A. Linear mixed model fit by maximum likelihood using the *lmer* function in R.

Formula: MC.MaxHR ~ (1 | Stream)

| AIC | BIC | logLik | deviance | df.resid |
| --- | --- | --- | --- | --- |
| 748.5 | 755.8 | -371.3 | 742.5 | 80 |

Scaled residuals:
Min 1Q Median 3Q Max

-2.6271 -0.6267 0.1662 0.7674 1.8486

Random effects:
Groups Name Variance Std.Dev.

Stream (Intercept) 61.32 7.831

Residual 424.26 20.598

Number of obs: 83, groups:  Stream, 3

 Fixed effects:
                    Estimate    Std. Error             t value
 (Intercept)  122.384      5.071       24.14

Table S4B. Coefficient estimates

Stream (Intercept) Estimate

Cyclone 121.0010

Langford 131.5430

McGee   114.6095

Table S5A. Linear mixed model fit by maximum likelihood using the *lmer* function in R.

Formula: Arr.Temp ~ (1 | Stream)

AIC BIC logLik deviance df.resid

414.3 421.6 -204.2 408.3 80

 Scaled residuals:
Min 1Q Median 3Q Max

-2.94756 -0.63214 0.08914 0.58530 1.74392

 Random effects:
  Groups        Variance Std.Dev.
  Stream  (Intercept) 0.5059   0.7113
  Residual         7.7274   2.7798

Number of obs: 83, groups:  Stream, 3

 Fixed effects:
         Estimate Std. Error t value
 (Intercept)  18.5511 0.5138 36.1

Table S5B: Coefficient estimates

Stream (Intercept) Estimate
 Cyclone 18.09371
 Langford 19.35222
 McGee   18.20723
